# Central Precocious Puberty and Psychiatric Disorders

**DOI:** 10.1001/jamanetworkopen.2025.16679

**Published:** 2025-06-23

**Authors:** Lars Dinkelbach, Corinna Grasemann, Cordula Kiewert, Lisa Leikeim, Börge Schmidt, Raphael Hirtz

**Affiliations:** 1 Department of Pediatrics III, University Hospital Essen, University of Duisburg-Essen, Essen, Germany; 2 Institute of Sex- and Gender-Sensitive Medicine, University Hospital Essen, University of Duisburg-Essen, Essen, Germany; 3 Division of Rare Diseases and Center for Rare Diseases Ruhr, Member of the European Reference Network on Rare Endocrine Conditions, Department of Pediatrics, St Josef-Hospital, Ruhr-University Bochum, Bochum, Germany; 4 Division of Pediatric Endocrinology and Diabetes, Member of the European Reference Network on Rare Endocrine Conditions, University Hospital Essen, University of Duisburg-Essen, Essen, Germany; 5 Health Data Lab, Gesellschaft für Wirtschaftlichkeit und Qualität bei Krankenkassen ServicePlus AG, Düsseldorf, Germany; 6 Institute of Medical Informatics, Biometry and Epidemiology, University Hospital Essen, University of Duisburg-Essen, Essen, Germany; 7 Center for Child and Adolescent Medicine, Helios University Hospital Wuppertal, Witten/Herdecke University, Wuppertal, Germany

## Abstract

**Importance:**

Within the physiological range, early pubertal timing is associated with an increased risk of mental health issues. Previous studies examining the associations of central precocious puberty (CPP) with mental health have yielded inconclusive results.

**Objective:**

To describe the risk for development of psychiatric disorders in patients with CPP and to identify periods during which patients with CPP are at heightened risk of developing psychopathological conditions.

**Design, Setting, and Participants:**

In this population-based, retrospective cohort study, patients with CPP and matched controls were identified from German health insurance data (approximately 6.5 million individuals) and followed from January 2010 to June 2023. Individuals were included if they had continuous insurance coverage for at least 2 years during the study period. Data were analyzed from July 2024 to March 2025.

**Exposure:**

Diagnosis of CPP.

**Main Outcomes and Measures:**

Diagnosis of depression, anxiety disorders, oppositional defiant and conduct disorders (ODD/CD), and attention deficit/hyperactivity disorder (ADHD). Incidence rates for psychiatric disorders before and after the diagnosis of CPP were compared between patients and controls exactly matched for sex, birth year interval, insurance period, and obesity.

**Results:**

After the application of validation criteria, 1094 patients with idiopathic CPP (438 born from 2010-2014 [40.0%]; 999 female [91.3%]; 249 [22.8%] with obesity) were identified and compared with 5448 controls (2184 born between 2010-2014 [40.1%]; 4975 female [91.3%]; 1242 with obesity [22.8%]). Compared with controls, patients with CPP were more likely to receive a diagnosis of any mental disorder (270 patients [24.7%] vs 920 controls [16.9%]; adjusted risk ratio [aRR], 1.48; 95% CI, 1.31-1.67), depression (82 patients [7.5%] vs 252 controls [4.6%]; aRR, 1.73; 95% CI, 1.37-2.20), anxiety disorders (88 patients [8.0%] vs 312 controls [5.7%]; aRR, 1.45; 95% CI, 1.16-1.82), ODD/CD (87 patients [8.0%] vs 243 controls [4.5%]; aRR, 1.76; 95% CI, 1.39-2.23), and ADHD (123 patients [11.2%] vs 397 controls [7.3%]; aRR, 1.53; 95% CI, 1.27-1.86). Temporal trends showed increased incidence rates for ODD/CD even before the diagnosis of CPP. For depression and ADHD, incidence rates remained increased for at least 8 years after the initial CPP diagnosis.

**Conclusions and Relevance:**

In this retrospective cohort study of patients with CPP, CPP was associated with an increased risk of psychiatric disorders, with evidence supporting long-term mental health outcomes, suggesting that caretakers of children with CPP should be vigilant for the emergence of psychiatric symptoms to initiate psychiatric care at an early stage.

## Key Points

**Question:**

Are patients with idiopathic central precocious puberty (CPP) at an elevated risk of developing psychiatric disorders?

**Findings:**

In this cohort study using health insurance data of 1094 patients with CPP and 5448 controls, patients with CPP exhibited a clinically relevant increased risk for developing depression, anxiety disorders, oppositional defiant and conduct disorders, and attention deficit/hyperactivity disorder, with elevated incidence rates persisting for at least 8 years after CPP diagnosis for certain conditions.

**Meaning:**

This study found evidence of an elevated risk of psychiatric disorders in patients with CPP, underscoring the importance of long-term psychiatric monitoring even years after the initial CPP diagnosis.

## Teaser

This cohort study analyzes associations of central precocious puberty with development of psychiatric disorders including depression, anxiety disorders, oppositional defiant and conduct disorders, and attention deficit/hyperactivity disorder among children in Germany.

## Introduction

Early puberty within the physiological range has consistently been associated with mental health issues, including an increased likelihood of developing depression, elevated levels of anxiety, attention deficits, a propensity for more aggressive and antisocial behaviors, and an elevated risk for substance abuse.^1,2,3,4,5,6^ Some studies indicate that the adverse effects of early puberty on mental health at least partially persist into early adulthood, extending beyond the typical period of peripubertal turbulence.^4,7^

Central precocious puberty (CPP) is characterized by a pathologically accelerated onset of pubertal development, defined by physical changes prior to the age of 8 years in girls (typically breast development) or prior to the age of 9 years in boys (typically testicular enlargement).^8^ CPP is approximately 9 times more frequent in girls than in boys and has shown a substantial increase in incidence rates in recent decades.^9^

Despite being one of the major concerns for parents seeking medical care for their child with presumed early or precocious pubertal development, the current knowledge about the psychosocial consequences of CPP is conflicting. Several smaller studies including up to 34 patients with CPP^10,11,12,13,14,15^ and 3 larger trials with up to 100 patients with CPP^16,17,18^ have compared the burden of psychopathological symptoms using standardized questionnaires against matched controls, normative values, or children with early but still physiologic pubertal timing. Notably, all studies^10,11,12,13,14,15,16,17,18^ were limited to female patients. In some of these studies, more internalizing (eg, depression or anxiety) and externalizing (eg, conduct disorders) symptoms were reported by patients with CPP or their parents,^10,11,12,13,18^ and at least some of this symptom burden persisted up to late adolescence.^12,13^ Other studies did not find evidence for more mental health issues in patients with CPP.^14,15,16,17^ Considering the moderate sample sizes, the diversity of study designs, methodological challenges (particularly a representative sampling of controls), and the divergent study results, recent reviews have argued that no clear conclusions can be drawn from the existing literature, thus identifying the psychological consequences of CPP as one of the most urgent topics for future research in this field.^8,19^ We aimed to (1) examine the association of idiopathic CPP with psychiatric disorders based on insurance data of approximately 6.5 million individuals and (2) describe the temporal association of the diagnosis of CPP with the diagnosis of psychiatric disorders.

## Methods

This cohort study was approved by the local ethics committee of the Medical Faculty of the University Duisburg-Essen. Given the anonymous nature of the utilized database, a waiver to obtain informed consent was granted by the ethics committee. The reporting of the study followed the Strengthening the Reporting of Observational Studies in Epidemiology (STROBE) reporting guideline.^26^

### Dataset Description

This study utilized an anonymized database of the Society for Efficiency and Quality in Health Insurance Funds (Gesellschaft für Wirtschaftlichkeit und Qualität bei Krankenkassen [GWQ]) ServicePlus AG, which integrates claims data of 19 statutory health insurances and covers approximately 6.5 million individuals throughout Germany. The anonymous database includes routinely collected demographic data, clinical diagnoses, and outpatient and inpatient care services. To ensure anonymity, the year of birth was assigned to intervals spanning 5-year periods (eg, 2005 to 2009). The utilized version of the dataset covered the period from January 1, 2010, to June 30, 2023 (ie, the analysis period).

In Germany, approximately 87% of the population is enrolled in statutory health insurances.^20^ Within this system, physicians document all patient contacts quarterly using *International Statistical Classification of Diseases and Related Health Problems, Tenth Revision (ICD-10)* codes and indicate diagnostic certainty (confirmed, suspected, excluded, or status post). For this study, only diagnoses classified as confirmed were considered.

### Inclusion and Exclusion Criteria

**Figure 1.**
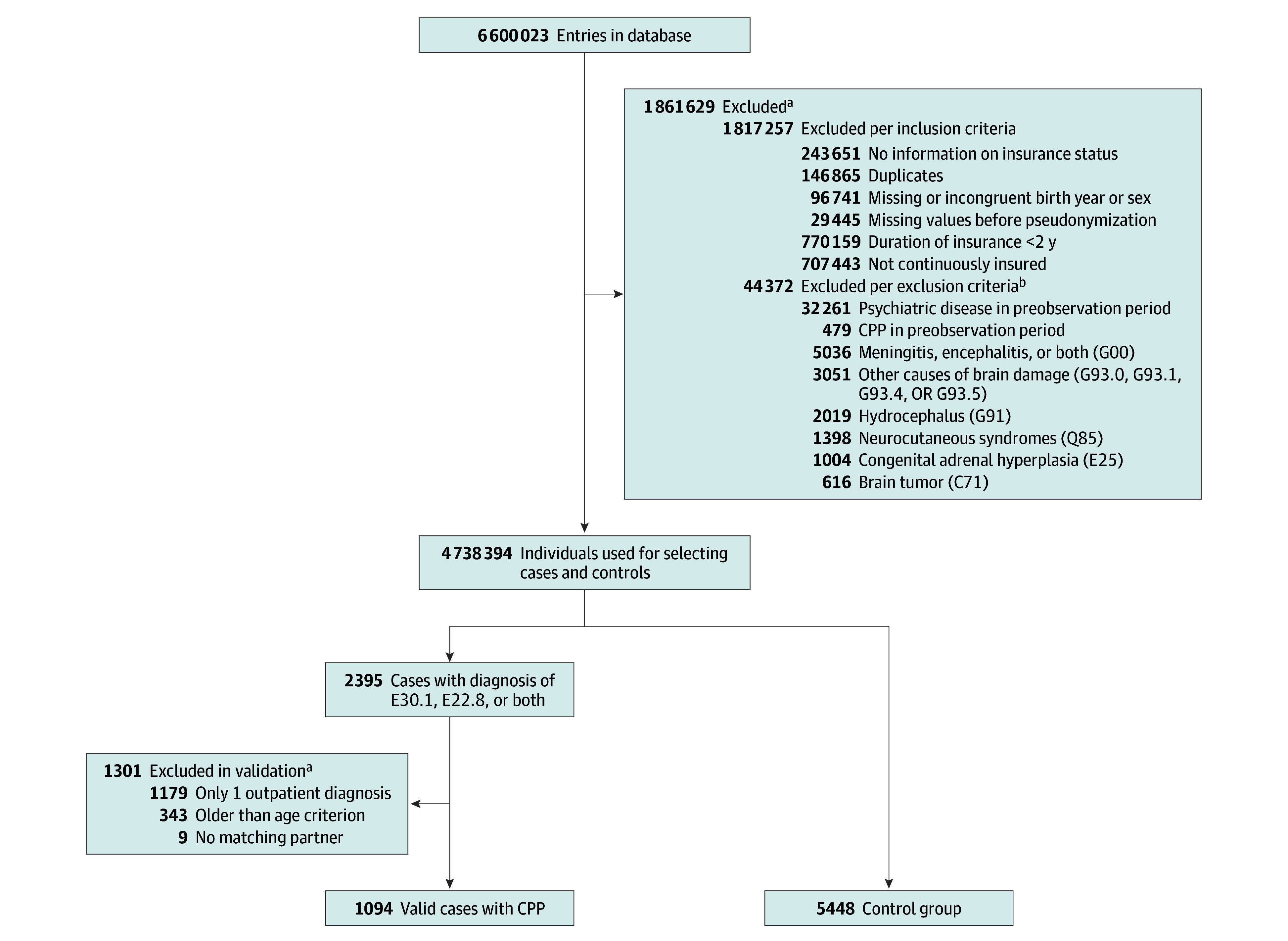
Identification of Cases and Controls This figure depicts the application of inclusion and exclusion criteria and identification of valid cases and controls, who were matched for birth year interval, sex, obesity, and insurance period. For each of the diagnoses, the respective *International Statistical Classification of Diseases and Related Health Problems, Tenth Revision (ICD-10) *codes are given. The preobservation period refers to the first year of documentation of insurance coverage (ie, either the year 2010, the first year after birth for persons born in 2010 or later, or, in cases with a change of insurance, the first year after enrollment in 1 of the insurance companies covered by the database). Due to the uncertainty of whether a coded diagnosis within the preobservation period referred to a new or preexisting condition, cases with either a coded diagnosis of central precocious puberty (CPP) or 1 of the psychiatric outcomes of interest within the preobservation period were excluded. Please note that the targeted 1:5 ratio between cases and controls was not fully achieved, with fewer than 5 matched controls available for 11 cases of CPP. ^a^Categories are not mutually exclusive. ^b^Due to restrictions of the database provider, the number of individuals with 1 of these diagnoses was limited to individuals born after the year 2000.

Given the nature of a routine database, duplicates and individuals with missing or inconsistent key data (insurance period, sex, and birth year) were excluded. The remaining individuals were included if they had continuous insurance coverage for at least 2 years during the analysis period. The first year of insurance coverage was defined as the preobservation period. Individuals who received a diagnosis of CPP or any psychiatric disorders of interest during the preobservation period were excluded. This exclusion criterion addressed uncertainty about whether diagnoses during the preobservation period represented new or preexisting conditions. Consequently, the observation period was defined as the time after the preobservation period until the end of insurance coverage or the end of the analysis period (June 30, 2023). Furthermore, individuals who received a diagnosis of conditions that may cause peripheral precocious puberty or secondary CPP^8,21^ (see Figure 1 for diagnoses and *ICD-10* codes used) were excluded. After applying these exclusion criteria, 4 738 394 individuals remained and were used to select the analysis population of idiopathic CPP cases and matched controls (Figure 1).

### Case Definition and Validation Criteria

CPP cases were identified by a diagnosis of either *ICD-10* codes E30.1 and/or E22.8 in the observation period, in accordance with the case definition utilized in previous epidemiological studies.^9,22^ Considering their prevalence during childhood and adolescence, the following psychiatric entities were included in the analysis: depression (*ICD-10* codes F32 and/or F33), anxiety disorders (*ICD-10* codes F40 or F41), oppositional defiant and conduct disorders (ODD/CD; *ICD-10* codes F91 and/or F90.1), attention deficit/hyperactivity disorder (ADHD; *ICD-10* codes F90 and/or F98.80), self-harming behavior (*ICD-10* code X84.9), and substance use disorders (*ICD-10* codes F10-F19).

Following a well-established validation strategy for secondary analyses of routine data^23,24^ and given the higher accuracy of inpatient diagnoses, an extended validation criterion was applied for case definition (for CPP or psychiatric disorders): only patients with a confirmed diagnosis on at least 2 occasions as an outpatient and/or 1 diagnosis as an inpatient qualified as a case. For CPP, an additional age-dependent validation criterion was applied: cases certainly older than 9 years (for girls) or older than 10 years (for boys) at first diagnosis were classified as implausible and disregarded as CPP cases. The age criterion was based on either the upper limit of the given birth year interval or the first quarter when the patient was insured. The age threshold of 9 years or younger for girls and 10 years or younger for boys has been used in previous large epidemiological studies on the secular trend of CPP and is based on the typical delay of about 6 to 12 months between the onset of symptoms and the first diagnosis of CPP.^9,22^

### Selection of Controls

For each identified CPP case, controls were exactly matched (on the individual level) for sex, birth year interval, insurance period, and whether obesity (*ICD-10* code E66) was diagnosed during the analysis period. For each CPP case, 5 matched controls were randomly selected (without replacement).

### Statistical Analysis

Data analysis was conducted from Data were analyzed from July 2024 to March 2025. In the main analysis, the probability that one of the psychiatric diseases of interest was diagnosed in the observation period (later referred to as the disease risk) was compared between CPP cases and matched controls using log-binomial regression models (R stats package^25^ version 4.3.2 [R Project for Statistical Computing]). We hypothesized that high health care utilization might lead to an increased identification of both CPP and psychiatric disorders, representing a potential confounding factor. Therefore, the number of routine child or youth examinations (*ICD-10* code Z00.1) was used as a surrogate marker for health care utilization and included as the only covariate in the regression models. Given that exact matching on the individual level for relevant confounders was achieved, we did not include these variables as covariates in the regression models.

To investigate the temporal association of the diagnosis of CPP with psychiatric diseases, the incidence rate per person-year at risk (ie, time within the preobservation period, an uninsured status, and time after a diagnosis of the outcome of interest did not contribute to the person-years at risk) for each year prior to or after the CPP diagnosis was calculated. Given the relatively limited number of cases observed each year, centered 3-year moving averages were computed, and incidence rate ratios were calculated to compare patients with CPP with controls. For the 3-year moving average, 95% CIs were determined under the assumption of a Poisson distribution (epitools package in R^49^ version 0.5-10.1). For the time periods earlier than 7 years before and later than 10 years after the first diagnosis of CPP, less than 20% of cases were still at risk, and thus the periods earlier than 7 years before and later than 10 years after the first diagnosis of CPP were aggregated.

#### Sensitivity and Exploratory Analyses

Several sensitivity analyses were conducted to evaluate whether specific choices of the case definition, additional covariates, or exclusion criteria biased the results. First, the analyses were reconducted without the extended validation criterion, meaning that a single outpatient or inpatient diagnosis was sufficient to qualify as a case. Second, we additionally adjusted for regional deprivation using the German Index of Socioeconomic Deprivation.^27^ Third and fourth, the occurrence of either traumatic brain injury (*ICD-10* code S06) or a diagnosis of thyroiditis or hypothyroidism (*ICD-10* codes E03 and/or E06) served as additional exclusion criteria. Fifth, sex-specific analyses were conducted to reveal possible sex-specific outcomes.

In exploratory analyses, we examined the directionality between CPP and psychiatric diagnoses. To assess whether prior psychopathology increases CPP risk, we analyzed a new sample without excluding individuals with preexisting psychiatric disorders in the preobservation period. Conversely, to evaluate the influence of preexisting psychopathology on the association of CPP with mental disorders, we analyzed only diagnoses occurring after CPP onset, adjusting for preexisting conditions. Finally, we investigated whether age at CPP diagnosis was associated with the risk of developing a mental disorder.

## Results

A total of 1094 validated CPP cases (438 born from 2010-2014 [40.0%]; 999 female [91.3%]; 249 [22.8%] with obesity) and 5448 matched controls (2184 born between 2010-2014 [40.1%]; 4975 female [91.3%]; 1242 with obesity [22.8%]) were included in the final cohort (Table). The number of CPP cases corresponded well to previously published incidence rates in a nationwide cohort study in Denmark^9^ (eAppendix 1 and eTable 1 in Supplement 1).

**Table.** Characteristics of the Total Cohort, Patients With CPP, and Matched Controls

Characteristics	Participants, No. (%)		
	Total sample (N = 6542)	CPP (n = 1094)	Matched controls (n = 5448)
Sex^a^			
Female	5974 (91.3)	999 (91.3)	4975 (91.3)
Male	568 (8.7)	95 (8.7)	473 (8.7)
Birth year			
2000-2004	1029 (15.7)	172 (15.7)	857 (15.7)
2005-2009	2261 (34.6)	379 (34.6)	1882 (34.5)
2010-2014	2622 (40.1)	438 (40.0)	2184 (40.1)
2015-2023^b^	630 (9.6)	105 (9.6)	525 (9.6)
Approx age at diagnosis, mean (SD), y^c^	NA	8.24 (2.0)	NA
Cases with obesity^d^	1491 (22.8)	249 (22.8)	1242 (22.8)
Time in the analysis period, mean (SD), m^e^	10.78 (3.0)	10.77 (3.0)	10.78 (3.0)
No. of routine child or youth examinations, mean (SD)^f^	3.87 (3.3)	4.07 (3.1)	3.83 (3.4)

Abbreviations: CPP, central precocious puberty; NA, not applicable.

^a^ Sex as documented from the insurance company.

^b^ Due to the database provider’s anonymity policies, birth years were grouped into 5-year intervals, and cases fewer than 5 were censored. To avoid censoring, the final interval (2020-2023) was combined with 2015-2019.

^c^ The age at diagnosis was approximated considering the restriction of the birth year to 5-year intervals.

^d^ Cases with documented obesity (*International Statistical Classification of Diseases and Related Health Problems, Tenth Revision [ICD-10] *code E66) during the analysis period.

^e^ Time within the analysis period from January 1, 2010, to June 30, 2023. Note that not all patients with CPP and controls were observed for the entire analysis period due to earlier discontinuation or later inclusion (eg, due to termination or change of insurance or later enrollment in one of the insurance companies covered by the database).

^f^ Number of routine child or youth examinations (*ICD-10* code Z00.1) during the analysis period.

### Risk for Psychiatric Disorders in Patients With CPP

**Figure 2.**
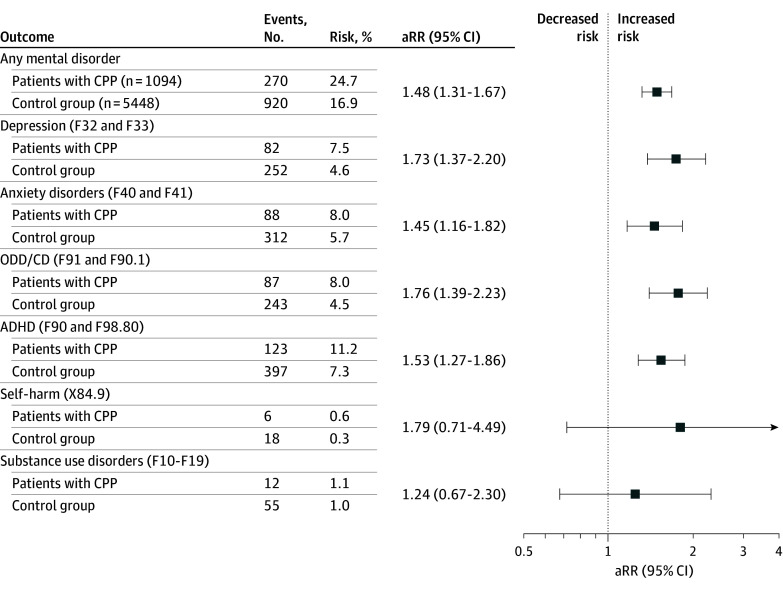
Risk for Psychiatric Disorders in Patients With Central Precocious Puberty (CPP) Compared With Controls The risk was defined by the probability that one of the psychiatric diseases of interest was diagnosed in the observation period. *International Statistical Classification of Diseases and Related Health Problems, Tenth Revision (ICD-10) *codes are given. The adjusted relative risk (aRR) refers to the results of the log-binomial regression model, including the number of routine child and youth examinations as a surrogate marker for health care utilization as a covariate. ADHD indicates attention deficit/hyperactivity disorder; ODD/CD, oppositional defiant and conduct disorders.

In comparison with matched controls, patients with CPP were more likely to develop depression (82 patients [7.5%] vs 252 controls [4.6%]; adjusted risk ratio [aRR], 1.73; 95% CI, 1.37-2.20), anxiety disorders (88 patients [8.0%] vs 312 controls [5.7%]; aRR, 1.45; 95% CI, 1.16-1.82), ODD/CD (87 patients [8.0%] vs 243 controls [4.5%]; aRR, 1.76; 95% CI, 1.39-2.23), and ADHD (123 patients [11.2%] vs 397 controls [7.3%]; aRR, 1.53; 95% CI, 1.27-1.86). As for self-harming behavior and substance use disorders, the number of incident cases was small, and the 95% CIs were wide and included the null effect (Figure 2). To give an estimate of the overall risk for any mental disorder, all valid cases of any one of the psychiatric diagnoses of interest (ie, F10-F19, F32-33, F40-41, F90-91, and F98.80) were considered, showing an overall increased risk for any mental disorder during the observation period of 24.7% in patients with CPP (270 patients) compared with 16.9% in controls (920 controls; aRR, 1.48, 95% CI, 1.30-1.67).

### Sensitivity and Exploratory Analyses

All sensitivity analyses generally confirmed the results (eAppendix 2 and eFigures 1-6 in Supplement 1), including an increased risk for mental disorders in male patients with CPP (risk for any mental disorder: 34 of 95 patients with CPP [35.8%] vs 100 of 473 controls [21.1%]; aRR, 1.69; 95% CI, 1.23-2.33). In exploratory analyses, documented mental disorders in the preobservation period were associated with increased CPP risk (aRR, 1.46; 95% CI, 1.08-1.97) (eFigure 7 in Supplement 1). After adjusting for preexisting psychopathology and examining only post-CPP diagnoses, CPP remained associated with a higher risk of depression, anxiety, ODD/CD, and ADHD (eFigure 8 in Supplement 1). There was no evidence that the approximated age at CPP diagnosis affected the risk of subsequent mental disorders (eFigure 9 in Supplement 1).

### Temporal Trends

**Figure 3.**
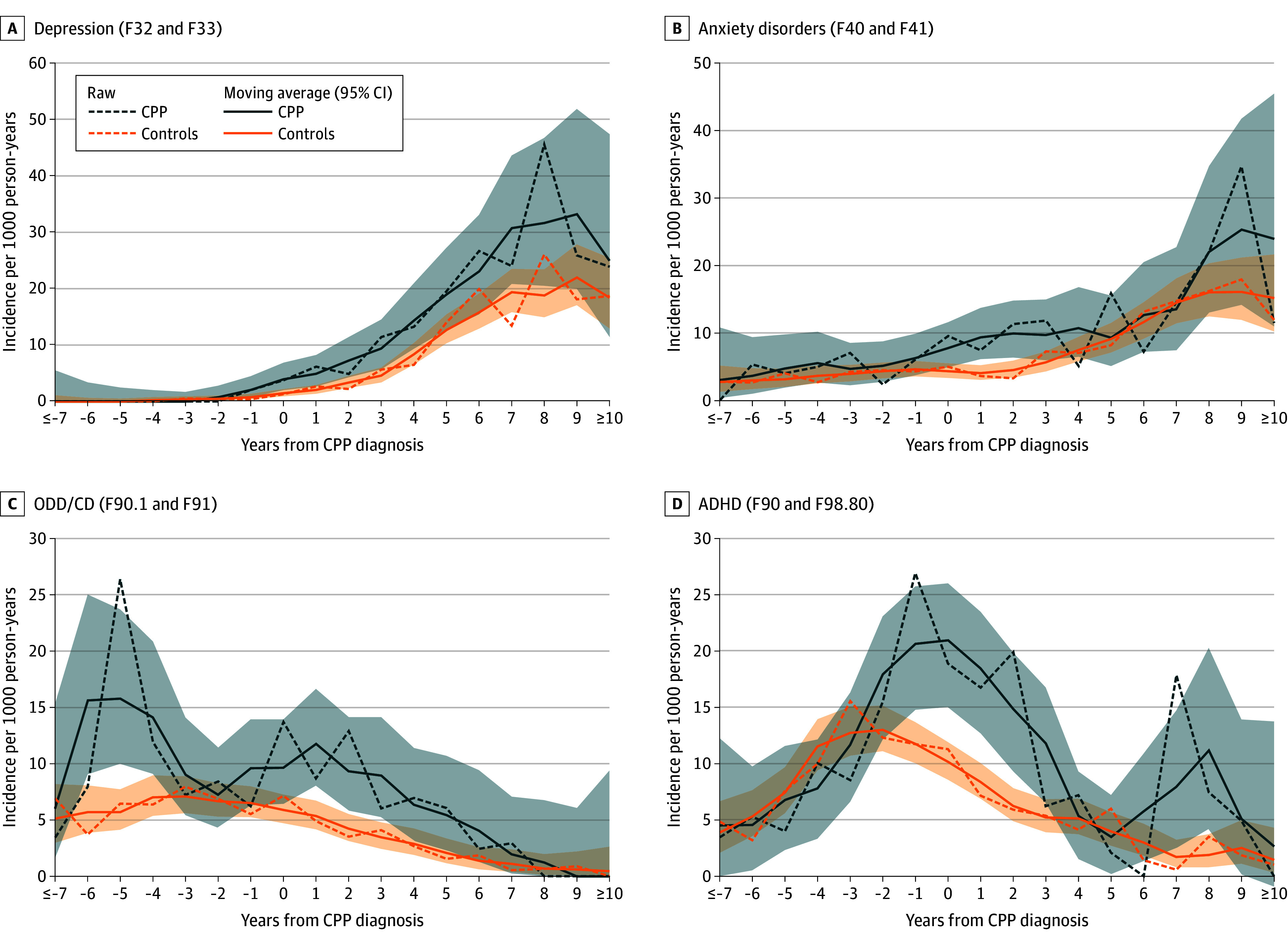
Temporal Trends of Incidence Rates of Psychiatric Diseases This figure illustrates the incidence rates per 1000 person-years for depression (A), anxiety disorders (B), oppositional defiant and conduct disorders (ODD/CD; C), and attention deficit/hyperactivity disorder (ADHD; D) for patients with central precocious puberty (CPP) and matched controls. *International Statistical Classification of Diseases and Related Health Problems, Tenth Revision (ICD-10) *codes are given. The dashed lines represent the raw incidence rates per year from the first diagnosis of CPP (ie, 2 years represents the incidence rate for the time frame from the ninth quarter to the 12th quarter after the quarter with the first CPP diagnosis). The solid line represents the 3-year centered moving average (ie, 2 years represents the incidence rate for the time frame from the fifth quarter to the 16th quarter after the first CPP diagnosis). The absolute numbers of events and person-years contributed by patients and controls are given in eTables 2 to 5 in Supplement 1. For incidence rate ratios comparing patients with CPP and controls, see eFigures 10 to 13 in Supplement 1.

To identify specific periods of risk, incidence rates (ie, newly diagnosed diseases per person-years at risk) for the psychiatric disorders were plotted against the time since or prior to the first diagnosis of CPP (Figure 3). Due to the limited number of cases, this analysis was not conducted for the outcomes of self-harm and substance use disorder. Using a centered 3-year moving average (ie, ±1 year for each time point), higher incidence rates in patients with CPP compared with controls were observed for ODD/CD up to 6 ± 1 years (due to a difference at 5 years), and for ADHD and depression up to 1 ± 1 years prior to CPP diagnosis. For all 4 analyzed outcomes (depression, anxiety disorders, ODD/CD, and ADHD), patients with CPP exhibited higher incidence rates around the time of CPP diagnosis (0 ± 1 year) (eTables 2-5 and eFigures 10-13 in Supplement 1). For depression and ADHD, higher incidence rates were observed up to 8 ± 1 years after the initial CPP diagnosis.

## Discussion

Previous studies on the consequences of CPP on mental health yielded conflicting results, and their informative value was partly limited due to small sample sizes, heterogeneous study designs, and methodological limitations. This large-scale retrospective cohort study, conducted in a population-based setting with controls randomly selected and individually matched for major confounders, provides evidence of an increased risk for patients with idiopathic CPP to develop depression, anxiety disorders, ODD/CD, and ADHD. Furthermore, this study found long-term negative mental health consequences in patients with CPP, with increased incidence rates for depression and ADHD persisting up to 8 ± 1 years after CPP diagnosis. These findings align with evidence from large-scale cohort studies suggesting an association of early pubertal timing within the physiological range with a wide spectrum of adverse mental health outcomes^1,2,3,4,7^ and support previous findings indicating that this association is long-lasting.^4,7^

Notably, the results of previous studies on the effects of early puberty within the physiological range have shown more inconsistencies in boys than in girls. A meta-analysis indicated adverse effects on mental health with early puberty in both sexes.^28^ However, recent epidemiologic studies, complemented by mendelian randomization studies, found an association of early puberty with depression in females but not in males.^2,5,29^ Regarding CPP, none of the studies to date, to our knowledge, included a male cohort. In the current study, sex-specific analyses demonstrated for the first time a higher risk of psychiatric disorders in boys with CPP, suggesting that the vulnerability to mental health in CPP affects both girls and boys.

Potential mechanisms underlying the association of CPP with a higher risk of psychiatric disorders include (1) physical and social changes resulting from early puberty, combined with age-related limitations in coping capacities, which may lead to psychological distress (ie, the maturation disparity hypothesis^30^). Bullying and harassment from peers may further aggravate this psychosocial distress, especially in girls.^17,31^ Alternatively, (2) endocrine changes associated with early puberty may lead to disrupted brain maturation or pathologically altered brain activity patterns (ie, the hormonal influence hypothesis^30^). The latter hypothesis is supported by the fact that puberty-related sex steroids directly interact with central nervous system receptors^32,33^ and are associated with adverse mental health outcomes.^34,35,36,37^ Lastly, (3) substantial preexisting psychological (ie, the accentuation hypothesis^30^) and social (ie, the contextual amplification hypothesis^30^) burdens may impair coping with early puberty and CPP, thereby exacerbating its negative effects on mental health.

Interestingly, we observed a higher incidence of ODD/CD well before CPP onset, consistent with a prior longitudinal study reporting early psychosocial difficulties in children who mature earlier than average.^38^ While this finding may reflect preexisting neurobiological (ie, the hormonal influence hypothesis) or psychosocial (ie, the accentuation hypothesis and contextual amplification hypothesis) risk factors, it also suggests that early mental health problems or adverse life events may contribute to early pubertal onset and progression, potentially leading to CPP. Supporting this theory, we found an increased risk of CPP in individuals with any psychiatric diagnosis during the preobservation period (eFigure 7 in Supplement 1). Prior studies have found associations of early adversity—particularly exposure to threat or abuse in infancy or early childhood—with accelerated pubertal timing.^39,40^ One proposed mechanism involves prolonged stress reducing cortisol’s inhibitory control over the hypothalamic-pituitary-gonadal axis, thereby triggering earlier puberty.^41^ Another possibility is that early adversity or psychopathology promotes social withdrawal and sedentary behavior, increasing obesity risk^42^—a known contributor to CPP.^8^ While early adversity or preexisting psychiatric conditions may contribute to CPP development, they likely do not fully explain the observed association of CPP with psychiatric disorders because the increased risk for mental disorders in patients with CPP persisted after controlling for psychiatric diagnoses prior to CPP diagnosis (eFigure 8 in Supplement 1). Together, these findings underscore a complex, bidirectional interplay among hormonal maturation, environmental factors, and mental health.

Our results demonstrate a clinically relevant association with poorer mental health outcomes in patients with CPP. One in 4 patients with CPP developed a psychiatric disease during the observation period, and approximately one-third of these diagnoses were not observed in matched controls. In previous studies, early pubertal timing within the physiologic range was not only associated with a higher risk for developing depression but also for recurring depressive episodes.^43^ Children and adolescents with behavioral or emotional problems are often underdiagnosed and untreated, despite detrimental effects on later life trajectories with an increased risk of poor academic achievement, unemployment, and premature death.^44,45,46^ Early intervention for mental health disorders in children and adolescents can alleviate disease burden and long-term negative psychosocial consequences.^47,48^ Thus, caretakers of patients with CPP should actively explore psychological symptoms and facilitate early intervention to influence lifetime trajectories of this vulnerable patient population positively. Because our findings indicate long-term sequelae of CPP on mental health, caretakers should be vigilant even after normalization of pubertal development.

### Limitations

The present study has several limitations. First, it relies on the accuracy of diagnoses by physicians in a population-based setting. Several steps have been taken to address this limitation: (1) Only diagnoses classified as confirmed by the caring physicians were considered, (2) only cases with at least 2 confirmed outpatient diagnoses (or 1 inpatient diagnosis) of an entity of interest were included in the study, and (3) for the CPP diagnosis, an additional age-dependent criterion was applied. Second, because the study employed routine data, confounding variables (eg, the socioeconomic status) could only be controlled to a limited extent. Therefore, our ability to draw conclusions about the directionality, causality, or underlying mechanisms of the association of CPP with mental health risk is limited. Third, using health insurance data necessitated strict adherence to anonymity policies, including grouping birth years into 5-year intervals. This constraint limited the precision of age-related analyses. Fourth, the limited number of cases with self-harming behaviors or substance use disorders in our study restricted our ability to draw conclusions about these risks in patients with CPP.

## Conclusions

To the best of our knowledge, this is the first cohort study (1) to provide evidence for an increased risk for psychiatric diseases in patients with CPP on the basis of more than 1000 patients with CPP, (2) to provide a comprehensive description of temporal patterns in this association, revealing an increased incidence rate for ODD/CD even prior to the onset of CPP and elevated incidence rates for depression and ADHD for at approximately 8 years after initial diagnosis, and (3) to describe an increased risk for mental disorders in males with CPP. Caretakers for children with CPP should be vigilant for the emergence of psychiatric symptoms, even at late follow-ups. Further research is required to elucidate the underlying pathophysiological mechanisms in order to develop efficacious treatments that can prevent the onset of psychiatric disorders in patients with CPP.

## Notes

Supplement 1. **eAppendix 1.** Comparison of the CPP Incidence Rate With Preexisting Literature **eTable 1.** CPP Incidence Rates **eAppendix 2.** Sensitivity Analyses **eFigure 1.** Results Without Extended Validation Criteria **eFigure 2.** Results With Additional Adjustment for Regional Deprivation Using the German Index of Socioeconomic Deprivation (GISD) **eFigure 3.** Results After Exclusion of Patients With Traumatic Brain Injuries **eFigure 4.** Results After Exclusion of Patients With Thyroiditis/Hypothyroidism **eFigure 5.** Results in Females Only **eFigure 6.** Results in Males Only **eFigure 7.** Risk for CPP After Diagnosis of Any Mental Disorder in the Pre-Observation Period **eFigure 8.** Risk for Psychiatric Disorders After CPP Diagnosis, Covarying for Preexisting Psychiatric Disorders **eFigure 9.** Effect of Age at CPP Diagnosis on the Risk to Develop Any Mental Disorder **eTable 2.** Depression: Cases and Person-Years at Risk Per Time Interval **eTable 3.** Anxiety Disorders: Cases and Person-Years at Risk Per Time Interval **eTable 4.** ODD/CD: Cases and Person-Years at Risk Per Time Interval **eTable 5.** ADHD: Cases and Person-Years at Risk Per Time Interval **eFigure 10.** Depression: Incidence Rate Ratios **eFigure 11.** Anxiety Disorders: Incidence Rate Ratios **eFigure 12.** ODD/CD: Incidence Rate Ratios **eFigure 13.** ADHD: Incidence Rate Ratios
eReferences.

Supplement 2.
Data Sharing Statement
