# Supplementary material for: Central Precocious Puberty and Psychiatric Disorders
Source: JAMA Netw Open. 2025 Jun 23;8(6):e2516679. doi: 10.1001/jamanetworkopen.2025.16679 (PMC12186511; doi:10.1001/jamanetworkopen.2025.16679)
Supplement: Supplement 1. — eAppendix 1. Comparison of the CPP Incidence Rate With Preexisting Literature eTable 1. CPP Incidence Rates eAppendix 2. Sensitivity Analyses eFigure 1. Results Without Extended Validation Criteria eFigure 2. Results With Additional Adjustment for Regional Deprivation Using the German Index of Socioeconomic Deprivation (GISD) eFigure 3. Results After Exclusion of Patients With Traumatic Brain Injuries eFigure 4. Results After Exclusion of Patients With Thyroiditis/Hypothyroidism eFigure 5. Results in Females Only eFigure 6. Results in Males Only eFigure 7. Risk for CPP After Diagnosis of Any Mental Disorder in the Pre-Observation Period eFigure 8. Risk for Psychiatric Disorders After CPP Diagnosis, Covarying for Preexisting Psychiatric Disorders eFigure 9. Effect of Age at CPP Diagnosis on the Risk to Develop Any Mental Disorder eTable 2. Depression: Cases and Person-Years at Risk Per Time Interval eTable 3. Anxiety Disorders: Cases and Person-Years at Risk Per Time Interval eTable 4. ODD/CD: Cases and Person-Years at Risk Per Time Interval eTable 5. ADHD: Cases and Person-Years at Risk Per Time Interval eFigure 10. Depression: Incidence Rate Ratios eFigure 11. Anxiety Disorders: Incidence Rate Ratios eFigure 12. ODD/CD: Incidence Rate Ratios eFigure 13. ADHD: Incidence Rate Ratios eReferences. [file jamanetwopen-e2516679-s001.pdf]

Supplemental Online Content

Dinkelbach L, Grasemann C, Kiewert C, Leikeim L, Schmidt B, Hirtz R. Central precocious puberty and psychiatric disorders. *JAMA Netw. Open*. 2025;8(6):e2516679. doi:10.1001/jamanetworkopen.2025.16679

eAppendix 1. Comparison of the CPP Incidence Rate With Preexisting Literature

eTable 1. CPP Incidence Rates

eAppendix 2. Sensitivity Analyses

eFigure 1. Results Without Extended Validation Criteria

eFigure 2. Results With Additional Adjustment for Regional Deprivation Using the German Index of Socioeconomic Deprivation (GISD)

eFigure 3. Results After Exclusion of Patients With Traumatic Brain Injuries

eFigure 4. Results After Exclusion of Patients With Thyroiditis/Hypothyroidism

eFigure 5. Results in Females Only

eFigure 6. Results in Males Only

eFigure 7. Risk for CPP After Diagnosis of Any Mental Disorder in the Pre-Observation Period

eFigure 8. Risk for Psychiatric Disorders After CPP diagnosis, Covarying for Preexisting Psychiatric Disorders

© 2025 Dinkelbach L et al. *JAMA Network Open*.

eFigure 9. Effect of Age at CPP Diagnosis on the Risk to Develop Any Mental Disorder

eTable 2. Depression: Cases and Person-Years at Risk Per Time Interval

eTable 3. Anxiety Disorders: Cases and Person-Years at Risk Per Time Interval

eTable 4. ODD/CD: Cases and Person-Years at Risk Per Time Interval

eTable 5. ADHD: Cases and Person-Years at Risk Per Time Interval

eFigure 10. Depression: Incidence Rate Ratios

eFigure 11. Anxiety Disorders: Incidence Rate Ratios

eFigure 12. ODD/CD: Incidence Rate Ratios

eFigure 13. ADHD: Incidence Rate Ratios

eReferences.

This supplemental material has been provided by the authors to give readers additional information about their work.

© 2025 Dinkelbach L et al. *JAMA Network Open*.

eAppendix 1. Comparison of the CPP Incidence Rate With Preexisting Literature

In a previous cohort study using national registry data in Denmark, the secular trend of a diagnosis of CPP was studied over a 20-year period between 1998 and 2017 [1]. In this study, a case of CPP was defined by at least one diagnosis of E30.1 or E22.8 before the age of nine in girls and before the age of ten in boys, similar to our study. The authors reported a total number of 6,856 cases (90.3% girls) of CPP in the observation period. Under the assumption of a relatively stable population of 5.8 million inhabitants in Denmark, this roughly translates to a yearly incidence rate of 59.1 CPP cases per one million inhabitants. A previous study of the same working group assessed the incidence of CPP (E30.1 or E22.8) between 1993 and 2001 using the same registry [2]. Here, medical records of 100 CPP cases were studied to verify the CPP diagnosis. Of these 100 cases, 46 had true precocious puberty, 36 had premature adrenarche or thelarche, and 14 had early pubertal development but within the age limits defining CPP [2]. Under the assumption of a similar ratio of 'true' CPP cases, this would translate to a yearly incidence rate of 27.2 'true' CPP cases per one million inhabitants in the later study Bräuner, Busch, Eckert-Lind, Koch, Hickey and Juul [1].

In our study, 2,203 cases of CPP (88.7% girls) were diagnosed between 2011 and 2022, translating to 49.7 CPP cases per one million insured individuals. Note that, in contrast to our main analyses, patients with comorbidities or psychiatric disorders in the pre-observation period were not excluded here to ensure comparability with the study by Bräuner, Busch, Eckert-Lind, Koch, Hickey and Juul [1]. By applying extended validation criteria, i.e., at least ≥ 2 outpatient diagnoses or one inpatient diagnosis of CPP, this incidence rate was reduced to 27.5 cases per one million insured individuals (for details, see Supplemental Table 1). Thus, the incidence rates of CPP in our study are comparable to the incidence rates reported by Bräuner, Busch, Eckert-Lind, Koch, Hickey and Juul [1], with a large overlap of the observation periods in both studies.

© 2025 Dinkelbach L et al. *JAMA Network Open*.

eTable 1. CPP Incidence Rates

		Without extended validation criterion [a]		With extended validation criterion [a]	
Year	Population [b]	Cases	Incidence per 10^6 per year	Cases	Incidence per 10^6 per year
2011	3,341,464	155	46.4	72	21.5
2012	3,488,491	225	64.5	117	33.5
2013	3,539,215	230	65.0	122	34.5
2014	3,580,180	162	45.2	98	27.4
2015	3,644,468	158	43.4	87	23.9
2016	3,724,960	163	43.8	78	20.9
2017	3,790,687	171	45.1	103	27.2
2018	3,821,785	181	47.4	100	26.2
2019	3,862,378	165	42.7	93	24.1
2020	3,869,009	196	50.7	104	26.9
2021	3,939,799	221	56.1	152	38.6
2022	3,847,647	176	45.7	98	25.5
		Mean (SD)	49.7 (7.9)		27.5 (5.4)

Average incidence rate of central precocious puberty (CPP) per million insured persons in a given year. [a] CPP cases without the extended validation criterion are individuals with a first diagnosis of either E30.1 or E22.8, excluding persons who do not meet the age criterion (i.e., girls older than nine years or boys older than ten years at the age of diagnosis), excluding persons with a CPP diagnosis in the pre-observation period (i.e., the first year of insurance), and excluding persons with missing or incongruent essential information or continuous insurance coverage less than two years. For the extended validation criterion, only individuals with ≥ 2 outpatient diagnoses of E30.1 or E22.8 or one inpatient diagnosis qualified as cases. [b] Here, 'population' refers to all individuals insured in the given year after excluding individuals with missing or incongruent essential information or an insufficient insurance period.

© 2025 Dinkelbach L et al. *JAMA Network Open*.

eAppendix 2. Sensitivity Analyses

To examine whether our findings are robust towards specific choices of inclusion or exclusion criteria, the following sensitivity analyses were conducted: 1.) Replication of the analysis without application of the extended validation criterion, 2.) after additional adjustment for regional deprivation using the German Index of Socioeconomic Deprivation (GISD), 3. and 4.) after additional exclusion of cases with traumatic brain injuries or hypothyroidism, as well as 5. and 6.) sex-specific analyses of either females or males only.

© 2025 Dinkelbach L et al. *JAMA Network Open*.

eFigure 1. Results Without the Extended Validation Criterion

Outcome	Events	Risk	adjusted RR (95% CI)
**Any mental disorder**			
CPP patients (N = 2,010)	709	35.27%	1.30 (1.22 to 1.40)
Controls (N = 10,015)	2722	27.18%	
**Depression (F32 and F33)**			
CPP patients	206	10.25%	1.37 (1.19 to 1.58)
Controls	775	7.74%	
**Anxiety disorders (F40 and F41)**			
CPP patients	298	14.83%	1.24 (1.10 to 1.40)
Controls	1214	12.12%	
**ODD/CD (F91 and F90.1)**			
CPP patients	249	12.39%	1.61 (1.41 to 1.85)
Controls	762	7.61%	
**ADHD (F90 and F98.80)**			
CPP patients	264	13.13%	1.35 (1.19 to 1.53)
Controls	972	9.71%	
**Self-harm (X84.9)**			
CPP patients	16	0.80%	1.38 (0.80 to 2.38)
Controls	61	0.61%	
**Substance use disorders (F10-F19)**			
CPP patients	36	1.79%	0.96 (0.68 to 1.36)
Controls	201	2.01%	

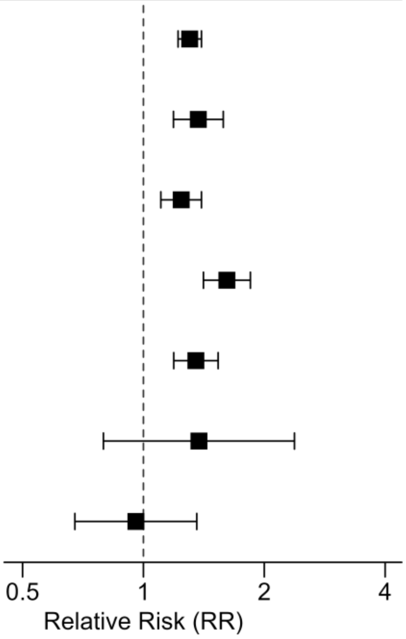

This figure illustrates the comparison of the risk (i.e., the probability of developing one of the psychiatric diseases of interest in the observation period between cases with central precocious puberty (CPP) and controls. For this sensitivity analysis, the extended validation criterion was not applied. Thus, each individual with a confirmed diagnosis of either CPP or one of the psychiatric diseases of interest qualified as a case. For CPP, only the age-criterion was applied as validation. The adjusted relative risk (RR) refers to the results of the log-binomial regression model, including the number of routine child/youth examinations as a surrogate marker for health care utilization as a covariate.

© 2025 Dinkelbach L et al. *JAMA Network Open*.

eFigure 2. Results With Additional Adjustment for Regional Deprivation Using the German Index of Socioeconomic Deprivation (GISD)

Outcome	Events	Risk	adjusted RR (95% CI)
**Any mental disorder**			
CPP patients (N = 1,094)	270	24.68%	1.46 (1.30 to 1.65)
Controls (N = 5,448)	920	16.89%	
**Depression (F32 and F33)**			
CPP patients	82	7.50%	1.71 (1.34 to 2.17)
Controls	252	4.63%	
**Anxiety disorders (F40 and F41)**			
CPP patients	88	8.04%	1.44 (1.14 to 1.80)
Controls	312	5.73%	
**ODD/CD (F91 and F90.1)**			
CPP patients	87	7.95%	1.75 (1.39 to 2.22)
Controls	243	4.46%	
**ADHD (F90 and F98.80)**			
CPP patients	123	11.24%	1.51 (1.25 to 1.84)
Controls	397	7.29%	
**Self-harm (X84.9)**			
CPP patients	6	0.55%	1.74 (0.69 to 4.37)
Controls	18	0.33%	
**Substance use disorders (F10-F19)**			
CPP patients	12	1.10%	1.20 (0.65 to 2.23)
Controls	55	1.01%	

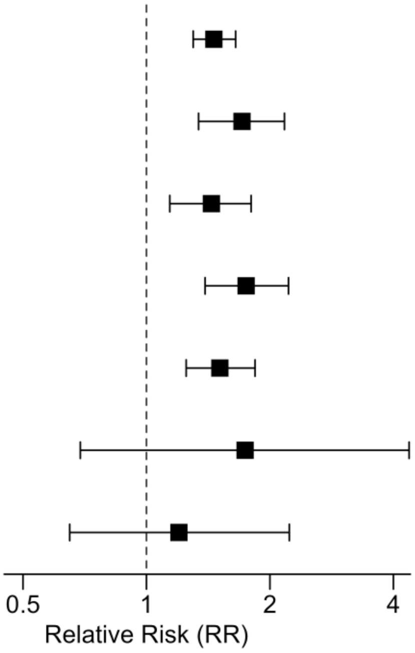

This figure illustrates the comparison of the risk (i.e., the probability of developing one of the psychiatric diseases of interest in the observation period between cases with central precocious puberty (CPP) and controls. For these sensitivity analyses, the regression model included the average German Index of Socioeconomic Deprivation (GISD) [3] of each CPP patient and the control's federal state to account for regional deprivation as a potential confounder. The GISD provides combined weighted (via principal component analyses) information on regional average income, employment rates, and educational levels. The average GISD for the 16 federal states was calculated by averaging the reported GISD scores across 38 NUTS-2 (Nomenclature of Territorial Units for Statistics – 2) regions for 2017, the midpoint of the observation period. Notably, this approach provided only a distant measure of individual deprivation or socioeconomic status and was therefore used solely in this sensitivity analysis.

© 2025 Dinkelbach L et al. *JAMA Network Open*.

eFigure 3. Results After Exclusion of Patients With Traumatic Brain Injuries

Outcome	Events	Risk	adjusted RR (95% CI)
**Any mental disorder**			
CPP patients (N = 1,007)	243	24.13%	1.54 (1.36 to 1.75)
Controls (N = 5,013)	795	15.86%	
**Depression (F32 and F33)**			
CPP patients	72	7.15%	1.71 (1.32 to 2.20)
Controls	222	4.43%	
**Anxiety disorders (F40 and F41)**			
CPP patients	82	8.14%	1.43 (1.13 to 1.81)
Controls	293	5.84%	
**ODD/CD (F91 and F90.1)**			
CPP patients	78	7.75%	1.77 (1.38 to 2.27)
Controls	218	4.35%	
**ADHD (F90 and F98.80)**			
CPP patients	113	11.22%	1.74 (1.42 to 2.13)
Controls	324	6.46%	
**Self-harm (X84.9)**			
CPP patients	5	0.50%	1.56 (0.58 to 4.23)
Controls	17	0.34%	
**Substance use disorders (F10-F19)**			
CPP patients	10	0.99%	1.09 (0.56 to 2.12)
Controls	51	1.02%	

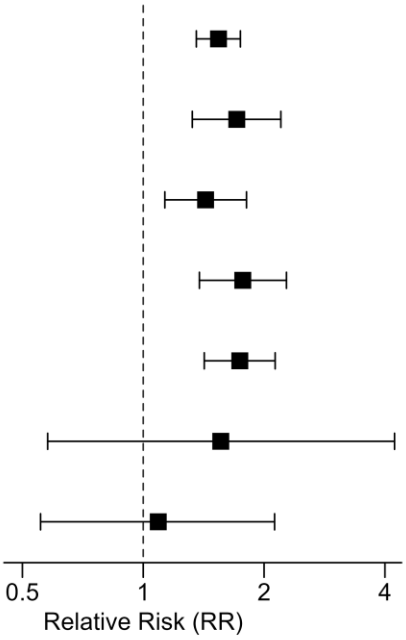

For these sensitivity analyses, all patients with a diagnosis of any traumatic brain injury (ICD-10 code S06) in the analysis period were excluded. Apart from this additional exclusion, all inclusion, exclusion, and validation criteria were consistent with those used in the main analyses. CPP = central precocious puberty; ICD-10 = International Statistical Classification of Diseases and Related Health Problems 10.

© 2025 Dinkelbach L et al. *JAMA Network Open*.

eFigure 4. Results After Exclusion of Patients With Thyroiditis/Hypothyroidism

Outcome	Events	Risk	adjusted RR (95% CI)
**Any mental disorder**			
CPP patients (N = 1,028)	241	23.91%	1.42 (1.25 to 1.61)
Controls (N = 5,020)	855	17.03%	
**Depression (F32 and F33)**			
CPP patients	70	6.94%	1.64 (1.27 to 2.12)
Controls	229	4.56%	
**Anxiety disorders (F40 and F41)**			
CPP patients	77	7.64%	1.51 (1.18 to 1.93)
Controls	262	5.22%	
**ODD/CD (F91 and F90.1)**			
CPP patients	79	7.84%	1.69 (1.32 to 2.16)
Controls	229	4.56%	
**ADHD (F90 and F98.80)**			
CPP patients	105	10.42%	1.38 (1.13 to 1.70)
Controls	378	7.53%	
**Self-harm (X84.9)**			
CPP patients	5	0.50%	1.54 (0.57 to 4.16)
Controls	17	0.34%	
**Substance use disorders (F10-F19)**			
CPP patients	10	0.99%	0.99 (0.51 to 1.93)
Controls	57	1.14%	

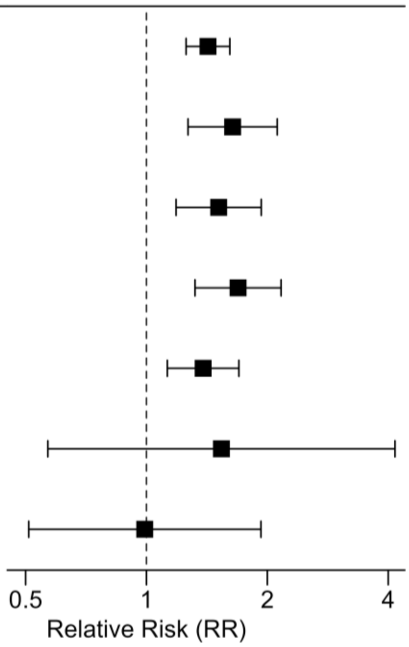

For these sensitivity analyses, all patients with a diagnosis of thyroiditis (ICD-10 code E06) or other hypothyroidism (ICD-10 code E03) in the analysis period were excluded. Besides this additional exclusion, all inclusion, exclusion, and validation criteria were consistent with those used in the main analyses. CPP = central precocious puberty; ICD-10 = International Statistical Classification of Diseases and Related Health Problems 10.

© 2025 Dinkelbach L et al. *JAMA Network Open*.

eFigure 5. Results in Females Only

Outcome	Events	Risk	adjusted RR (95% CI)
**Any mental disorder**			
CPP patients (N = 999)	236	23.62%	1.48 (1.30 to 1.68)
Controls (N = 4,975)	808	16.24%	
**Depression (F32 and F33)**			
CPP patients	76	7.61%	1.72 (1.34 to 2.20)
Controls	234	4.70%	
**Anxiety disorders (F40 and F41)**			
CPP patients	81	8.11%	1.37 (1.08 to 1.73)
Controls	304	6.11%	
**ODD/CD (F91 and F90.1)**			
CPP patients	71	7.11%	1.97 (1.51 to 2.57)
Controls	178	3.58%	
**ADHD (F90 and F98.80)**			
CPP patients	100	10.01%	1.62 (1.31 to 2.01)
Controls	309	6.21%	
**Self-harm (X84.9)**			
CPP patients	6	0.60%	2.86 (1.06 to 7.72)
Controls	11	0.22%	
**Substance use disorders (F10-F19)**			
CPP patients	11	1.10%	1.49 (0.77 to 2.88)
Controls	41	0.82%	

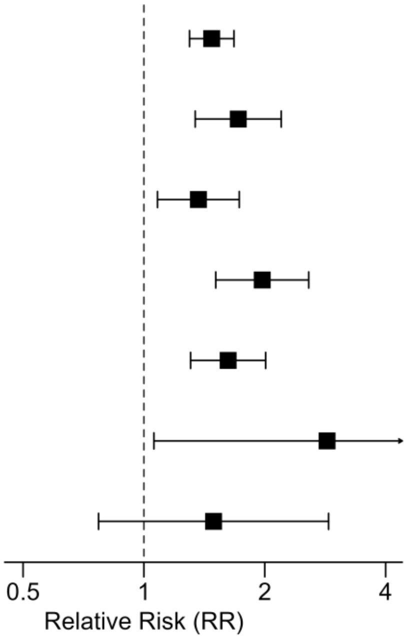

For these sensitivity analyses, only female patients with central precocious puberty (CPP) and their respective controls were considered. All inclusion, exclusion, and validation criteria were consistent with those used in the main analyses. In contrast to the primary analysis, female patients exhibited a heightened propensity for self-harming behaviors when evaluated under conventional statistical significance criteria. However, the number of events was low, and the significant finding observed in comparison to the primary analysis is likely attributable to the new sampling of random controls. Therefore, this finding should not be interpreted as a sex-specific effect, and further research is required to elucidate the consequences of CPP for self-harming behavior.

© 2025 Dinkelbach L et al. *JAMA Network Open*.

eFigure 6. Results in Males Only

Outcome	Events	Risk	adjusted RR (95% CI)
**Any mental disorder**			
Patients with CPP (N = 95)	34	35.79%	1.69 (1.23 to 2.33)
Controls (N = 473)	100	21.14%	

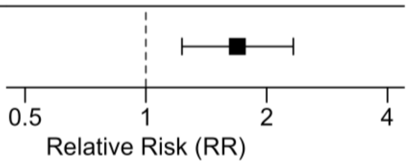

For these sensitivity analyses, only male patients with central precocious puberty (CPP) and their respective controls were considered. All inclusion, exclusion, and validation criteria, as in the main analyses, were applied. Given the limited number of male patients in our study, only the overall incidence of any mental disorder in the observation period was examined.

© 2025 Dinkelbach L et al. *JAMA Network Open*.

eFigure 7. Risk for CPP After Diagnosis of Any Mental Disorder in the Pre-Observation Period

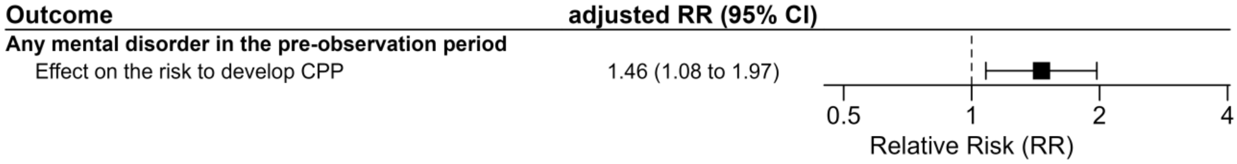

In the main analysis, the presence of any mental disorder in the pre-observation period was an exclusion criterion. To address whether earlier psychopathologies influenced the risk of developing precocious puberty (CPP), we conducted a separate analysis using a new sample without this exclusion criterion, resulting in N = 1,130 CPP patients and N = 5,626 randomly selected, matched controls (matched for sex, birth-year interval, insurance period, and obesity). Here, the number of cases with any of the psychiatric diagnoses during the pre-observation period was 3.1% (N = 35) for CPP patients and 2.1% (N = 118) for controls. In this newly defined sample, a psychiatric diagnosis during the pre-observation period was identified as a risk factor for the subsequent development of CPP (RR = 1.46, 95% CI [1.08, 1.97]).

© 2025 Dinkelbach L et al. *JAMA Network Open*.

eFigure 8. Risk for Psychiatric Disorders After CPP Diagnosis, Covarying for Preexisting Psychiatric Disorders

Outcome	Events	Risk	adjusted RR (95% CI)
**Any mental disorder**			
CPP patients (N = 1,094)	193	17.64%	1.46 (1.26 to 1.69)
Controls (N = 5,448)	655	12.02%	
**Depression (F32 and F33)**			
CPP patients	80	7.31%	1.66 (1.31 to 2.12)
Controls	246	4.52%	
**Anxiety disorders (F40 and F41)**			
CPP patients	68	6.22%	1.52 (1.03 to 2.25)
Controls	230	4.22%	
**ODD/CD (F91 and F90.1)**			
CPP patients	45	4.11%	2.00 (1.42 to 2.83)
Controls	107	1.96%	
**ADHD (F90 and F98.80)**			
CPP patients	67	6.12%	1.44 (1.10 to 1.87)
Controls	228	4.18%	
**Self-harm (X84.9)**			
CPP patients	6	0.55%	1.77 (0.69 to 4.49)
Controls	17	0.31%	
**Substance use disorders (F10-F19)**			
CPP patients	11	1.00%	1.10 (0.58 to 2.09)
Controls	54	0.99%	

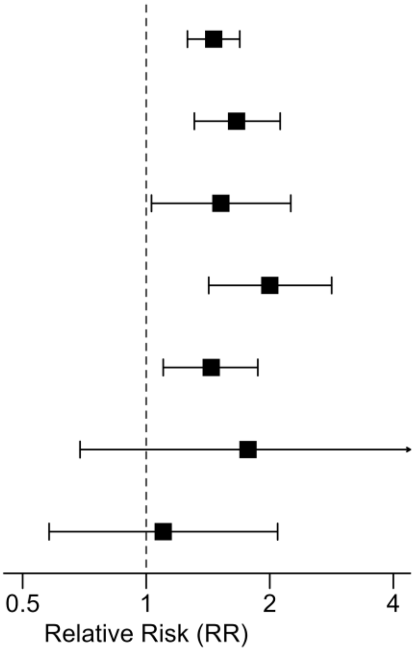

For these sensitivity analyses, only psychiatric disorder diagnoses first recorded after or within the same quartal as the initial diagnosis of central precocious puberty (CPP) were considered. Additionally, a covariate was included to indicate whether a CPP patient or control had any psychiatric diagnosis prior to CPP onset. While this approach resulted in slightly lower effect estimates across all diagnoses, the overall pattern remained consistent with the main analysis.

© 2025 Dinkelbach L et al. *JAMA Network Open*.

eFigure 9. Effect of Age at CPP Diagnosis on the Risk to Develop Any Mental Disorder

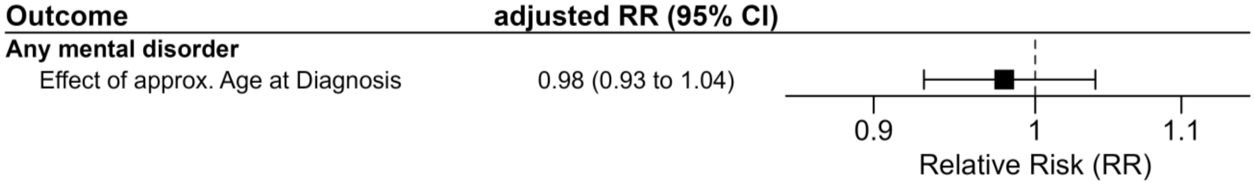

To comply with the anonymity guidelines set by the database provider, birth years for both cases and controls were provided only in five-year intervals. As a result, precise conclusions regarding the exact age at diagnosis of central precocious puberty (CPP) were limited. To examine the impact of age at diagnosis on the risk of developing psychopathologies in CPP patients, an approximation was made by calculating the mean of the minimum and maximum possible ages within each five-year interval. This approach yielded an estimated mean age at diagnosis of 8.24 years (± 1.99). The approximated age at diagnosis was then incorporated into a log-binomial regression model, including sex, the length of the insurance period, the number of routine child or youth examinations (ICD-10: Z00.1) and whether obesity (ICD-10: E66) was diagnosed during the analysis period as covariates. This analysis was conducted exclusively in CPP patients (N = 1,094).

© 2025 Dinkelbach L et al. *JAMA Network Open*.

eTable 2. Depression: Cases and Person-Years at Risk Per Time Interval

	Years from CPP diagnosis					
	≤-5	-4 to -2	-1 to +1	+2 to +4	+5 to +7	≥ +8
**CPP patients**						
Events	0	0	12	20	29	21
PY at Risk	1,171	2,186	3,036	2,129	1,259	624
**Controls**						
Events	0	≤5	22	49	104	73
PY at Risk	5,842	10,899	15,141	10,717	6,580	3,380

This table depicts the number of events, i.e., the occurrence of depression and the person-years (PY) at risk contributed by patients with central precocious puberty (CPP) and controls. Due to the anonymity policies of the database provider, the time frames were aggregated in three-year periods, and cases ≤ 5 were censored.

© 2025 Dinkelbach L et al. *JAMA Network Open*.

eTable 3. Anxiety Disorders: Cases and Person-Years at Risk Per Time Interval

	Years from CPP diagnosis					
	≤-5	-4 to -2	-1 to +1	+2 to +4	+5 to +7	≥ +8
**CPP patients**						
Events	≤5	10	23	20	16	15
PY at Risk	1,167	2,160	2,977	2,063	1,268	648
**Controls**						
Events	19	42	64	59	75	53
PY at Risk	5,819	10,802	14,910	10,476	6,469	3,398

This table depicts the number of events, i.e., the occurrence of an anxiety disorder and the person-years (PY) at risk contributed by patients with central precocious puberty (CPP) and controls. Due to the anonymity policies of the database provider, the time frames were aggregated in three-year periods, and cases ≤ 5 were censored.

© 2025 Dinkelbach L et al. *JAMA Network Open*.

eTable 4. ODD/CD: Cases and Person-Years at Risk Per Time Interval

	**Years from CPP diagnosis**					
	≤-5	-4 to -2	-1 to +1	+2 to +4	+5 to +7	≥ +8
**CPP patients**						
Events	17	19	28	18	≤5	0
PY at Risk	1,161	2,107	2,906	2,013	1,238	667
**Controls**						
Events	33	76	87	36	9	≤5
PY at Risk	5,802	10,710	14,741	10,377	6,535	3,566

This table depicts the number of events, i.e., the occurrence of any oppositional defiant or conduct disorder (ODD/CD), and the person-years (PY) at risk contributed by patients with central precocious puberty (CPP) and controls. Due to the anonymity policies of the database provider, the time frames were aggregated in three-year periods, and cases ≤ 5 were censored.

© 2025 Dinkelbach L et al. *JAMA Network Open*.

eTable 5. ADHD: Cases and Person-Years at Risk Per Time Interval

	Years from CPP diagnosis					
	≤-5	-4 to -2	-1 to +1	+2 to +4	+5 to +7	≥ +8
**CPP patients**						
Events	≤5	25	60	23	7	≤5
PY at Risk	1,166	2,144	2,869	1,951	1,220	648
**Controls**						
Events	32	137	148	53	19	8
PY at Risk	5,827	10,763	14,623	10,158	6,348	3,469

This table depicts the number of events, i.e., the occurrence of an attention deficit hyperactivity disorder (ADHD) and the person-years (PY) at risk contributed by patients with central precocious puberty (CPP) and controls. Due to the anonymity policies of the database provider, the time frames were aggregated in three-year periods and, cases ≤ 5 were censored.

© 2025 Dinkelbach L et al. *JAMA Network Open*.

e**Figure 10**. **Depression: Incidence** Rate Ratios

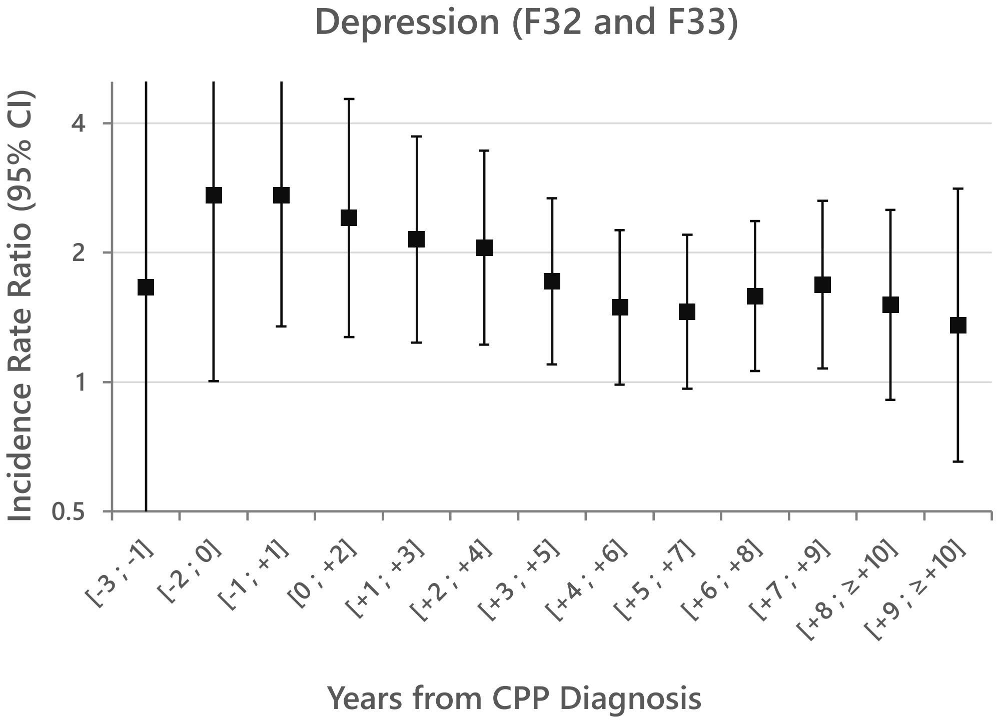

Incidence rate ratios > 1 indicate higher incidence rates in CPP patients compared to matched controls. Incidence rate ratios for three-year moving averages were calculated by unconditional maximum likelihood estimation with normal approximation (Wald). Error bars represent 95% confidence intervals (95% CI).

© 2025 Dinkelbach L et al. *JAMA Network Open*.

eFigure 11. Anxiety Disorders: Incidence Rate Ratios

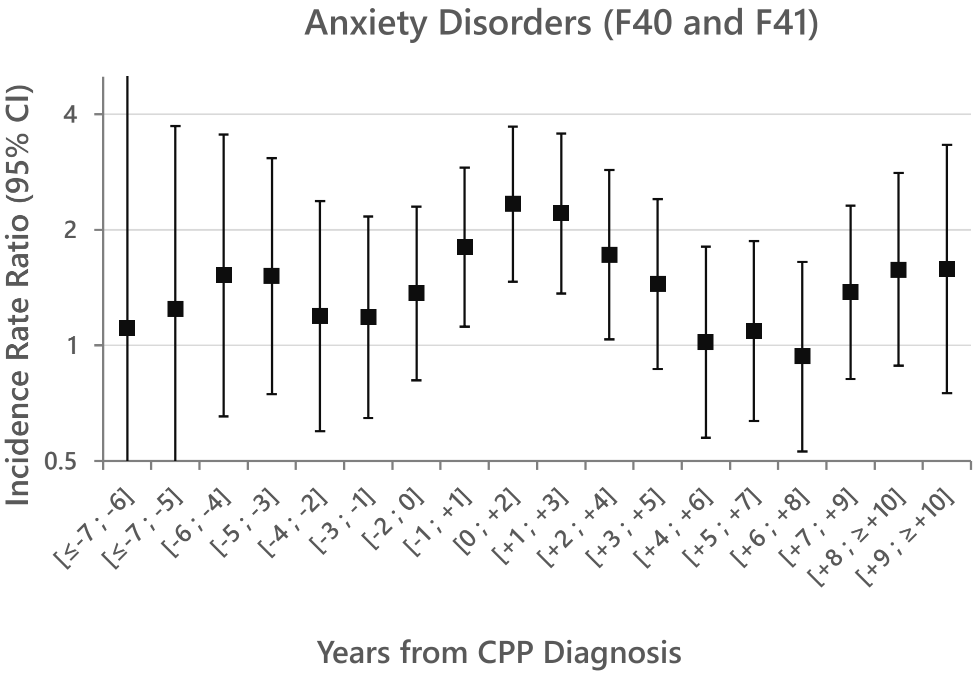

Incidence rate ratios > 1 indicate higher incidence rates in CPP patients compared to matched controls. Incidence rate ratios for three-year moving averages were calculated by unconditional maximum likelihood estimation with normal approximation (Wald). Error bars represent 95% confidence intervals (95% CI).

© 2025 Dinkelbach L et al. *JAMA Network Open*.

eFigure 12. ODD/CD: Incidence Rate Ratios

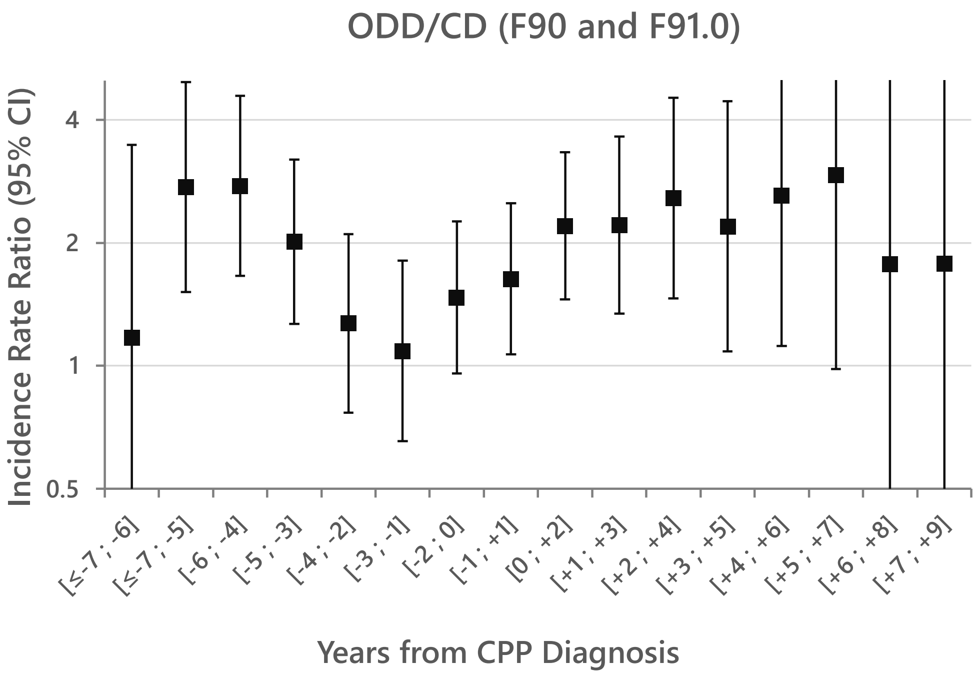

Incidence rate ratios > 1 indicate higher incidence rates in CPP patients compared to matched controls. Incidence rate ratios for three-year moving averages were calculated by unconditional maximum likelihood estimation with normal approximation (Wald). Error bars represent 95% confidence intervals (95% CI).

© 2025 Dinkelbach L et al. *JAMA Network Open*.

eFigure 13. ADHD: Incidence Rate Ratios

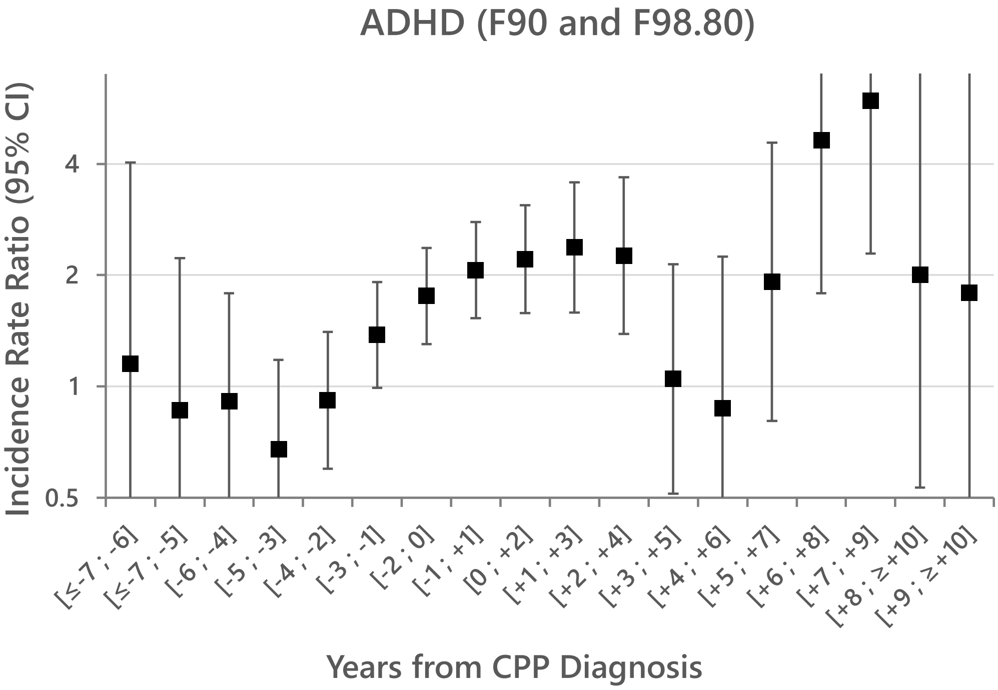

Incidence rate ratios > 1 indicate higher incidence rates in CPP patients compared to matched controls. Incidence rate ratios for three-year moving averages were calculated by unconditional maximum likelihood estimation with normal approximation (Wald). Error bars represent 95% confidence intervals (95% CI).

© 2025 Dinkelbach L et al. *JAMA Network Open*.

eReferences

1. Bräuner EV, Busch AS, Eckert-Lind C, Koch T, Hickey M, Juul A. Trends in the incidence of central precocious puberty and normal variant puberty among children in Denmark, 1998 to 2017. *JAMA network open*. 2020;3(10):e2015665-e2015665.

2. Teilmann G, Pedersen CB, Jensen TK, Skakkebæk NE, Juul A. Prevalence and incidence of precocious pubertal development in Denmark: an epidemiologic study based on national registries. *Pediatrics*. 2005;116(6):1323-1328.

3. Michalski N, Reis M, Tetzlaff F, et al. German Index of Socioeconomic Deprivation (GISD): revision, update and applications. *Journal of health monitoring*. 2022;7(Suppl 5):2.

© 2025 Dinkelbach L et al. *JAMA Network Open*.
