# Supplementary material for: Central Precocious Puberty and Psychiatric Disorders
Source: JAMA Netw Open. 2025 Jun 23;8(6):e2516679. doi: 10.1001/jamanetworkopen.2025.16679 (PMC12186511; doi:10.1001/jamanetworkopen.2025.16679)
Supplement: Supplement 2. — Data Sharing Statement [file jamanetwopen-e2516679-s002.pdf]

Data Sharing Statement

Dinkelbach. Central Precocious Puberty and Psychiatric Disorders. *JAMA Netw Open*. Published June 23, 2025. doi:10.1001/jamanetworkopen.2025.16679

Data
Data available: No

Additional Information
Explanation for why data not available: Due to legal regulations of the database provider, the primary data of the study cannot be shared publicly.
